# First case of bronchiolar adenoma lined purely by mucinous luminal cells with molecular analysis: A case report

**DOI:** 10.1097/MD.0000000000022322

**Published:** 2020-09-25

**Authors:** Shuli Liu, Nan Liu, Mingming Xiao, Liang Wang, En-Hua Wang

**Affiliations:** a Department of Pathology, the First Affiliated Hospital and College of Basic Medical Sciences, China Medical University; b Department of Pathology, the People's Hospital of Liaoning Province, Shenyang, China.

**Keywords:** ALK, BRAF, bronchiolar adenoma, ciliated muconodular papillary tumor, immunohistochemistry

## Abstract

**Rationale:**

Bronchiolar adenoma (BA) is a newly designated rare entity of the lung, including both the currently designated ciliated muconodular papillary tumor (CMPT) and so-called non-classic CMPT. The most prominent histological feature of BAs is the bilayered cell structures composed of the continuous basal cell layer and the luminal layer which consists of different proportion of mucinous cells, ciliated cells, Clara cells and/or type II pneumocytes. BA purely covered by mucinous cells without other components in the luminal layer has never been reported.

**Patient concerns:**

An 82-year-old female patient was detected a 0.8 cm ground glass nodule in the left lower lobe of the lung.

**Diagnoses:**

The serum levels of tumor markers were normal.

**Interventions:**

The patient underwent a segmentectomy of the left lower lobe.

**Outcomes:**

The postoperative pathological diagnosis was BA. Molecular analysis revealed that the tumor harbored ALK rearrangement and BRAF mutations simultaneously. There was no recurrence in 17 months of follow-up.

**Lessons:**

BA can be lined only by mucinous cells, without any cuboidal and/or ciliated cells in the surface layer. This sets a dangerous pitfall in differentiation diagnosis with invasive mucinous adenocarcinoma especially during intraoperative frozen pathological diagnosis.

## 1 Introduction

Bronchiolar adenoma (BA) is a newly designated entity of benign lung tumor which will be recognized in the upcoming World Health Organization classification in 2020. This entity encompass a spectrum of lesions including both the currently designated ciliated muconodular papillary tumor (CMPT) and so-called non-classic CMPT. The most prominent histological feature of BA is the bilayered cell structures composed of the continuous basal cell layer and the luminal cell layer which consists of different proportion of mucinous cells, ciliated cells, Clara cells and/or type II pneumocytes.^[1]^ Based on the composition of surface cells, BA can be further divided into proximal-type BA and distal-type BAs. The proximal type is lined by ciliated and mucinous cells, in papillary or flat pattern. The distal type is generally flatted, covered by mucinous cells, cuboidal and/or ciliated cells. For this rare entity, the distal-type BA purely covered by mucinous cells has never been reported. This unique and extremely rare case can be easily misdiagnosed as invasive mucinous adenocarcinoma (IMA). Herein, we report this case with a focus of immunohistochemical features and driver gene mutations.

## 2 Case report

A 81-year-old female patient was initially detected a 0.6 cm ground glass nodule in the left lower lobe of the lung by computed tomography (CT) during healthy examination in June 2017. The nodule grew to 0.8 cm in August 2018, and no enlarged hilar and mediastinal lymph nodes were identified. The serum levels of tumor markers, including carcinoembryonic antigen, neuron-specific enolase, cytokeratin-19 fragment, etc. were normal. The patient underwent a segmentectomy of the left lower lobe, and macroscopical examination revealed a gray-white mucoid mass, measuring 1.0 × 0.6 cm. An intraoperative frozen pathological diagnosis favored benign mucinous tumor, and deferred to permanent sections to rule out IMA.

**Figure 1:**
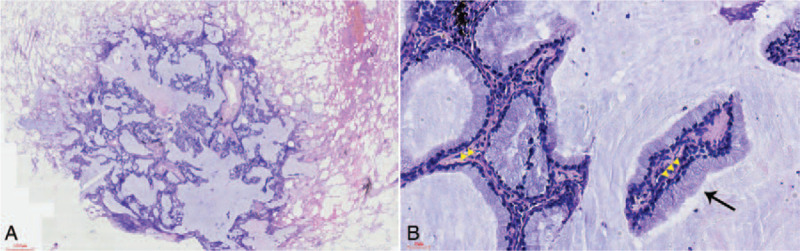
The tumor cells grew along the alveolar walls, with a small number of papillary and adenoid structures, with abundant extracellular mucin (A, 40×). The surface-layered tumor cells were purely tall columnar cells (B, 400×, black arrow) with areas showing bilayered structure (yellow arrowhead) with a continuous basal cell layer.

Postoperative pathological examination showed the well-circumscribed tumor is located in the lung parenchyma with a maximum diameter of 1.0 cm. The tumor cells grew along the alveolar walls, with a small number of papillary and adenoid structures (Fig. 1A). The luminal tumor cells were purely mild and tall columnar mucinous cells. No ciliated or cuboidal cells could be evident. The nuclei were located in the basal part, which seemed to be arranged in a single layer. However, in certain areas, the tumor was composed of bilayered cellular proliferation with a continuous basal cell layer (Fig. 1B).

**Figure 2:**
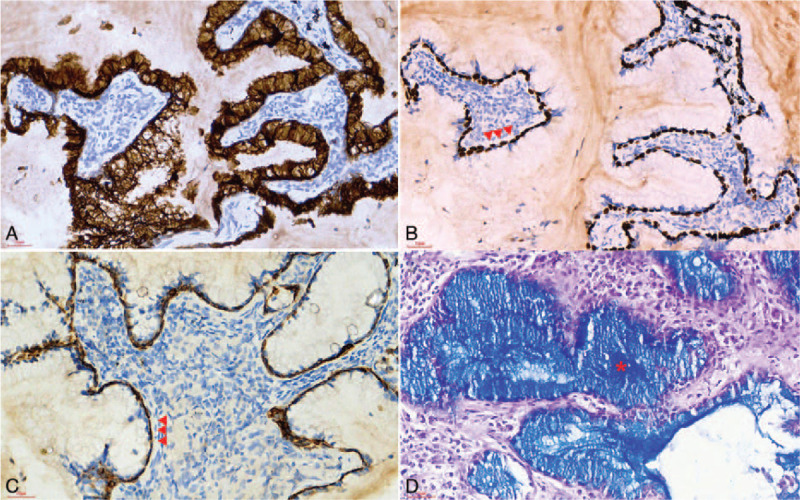
The surface-layered tumor cells were positive for CK7 (A, 400×). The basal-layered tumor cells showed continuous positive staining for p63 (B, 400×, arrowhead) and CK5/6 (C, 400×, arrowhead). AB-PAS demonstrated abundant intracellular and extracellular mucin (D, 400×, asterisk).

**Figure 3:**
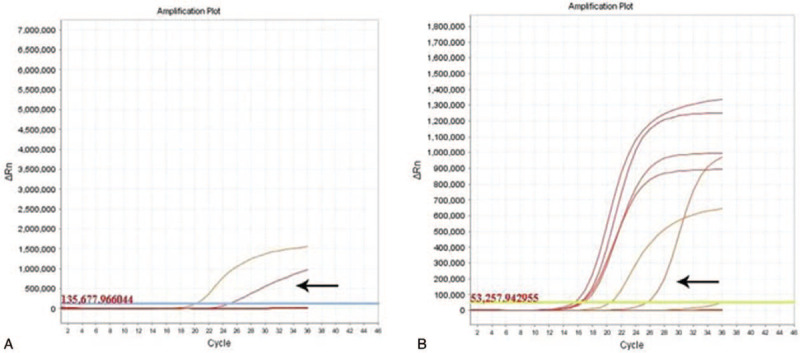
Quantitative reverse-transcript Polymerase chain reaction (qRT-PCR) revealed *ALK* rearrangement (A, arrow) and *BRAF* mutations (B, arrow) in the tumor cells.

Immunohistochemically, the columnar luminal cells were strongly positive for CK7 (Fig. 2A), but negative for thyroid transcription factor-1 (TTF-1). Staining for CK5/6, p40, p63 and TTF-1 revealed an intact and continuous bottom layer of basal cells (Fig. 2B and 2C). The index of Ki-67 was less than 1%. The basal cells were negative for myoepithelial markers, such as S-100, smooth muscle antibody (SMA), and CD117. Alcian blue/periodic acid–Schiff (AB-PAS) staining demonstrated abundant mucin in the cytoplasm of columnar cells and extracellular spaces (Fig. 2D). The final histological diagnosis was rendered as BA. A small panel of driver genes were examined using the ADx-ARMS Kit (Amoy Diagnostics, Xiamen, China). Rearrangement of *ALK* (Fig. 3A) and mutations of *BRAF* (Fig. 3B) were identified. The post-operative course was uneventful, and no recurrence was noted at 17 months’ follow-up.

## 3 Discussion

In 2002, Ishikawa^[2]^ first, described and reported a rare tumor in the peripheral lung and named it as CMPT. CMPT is easily to be misdiagnosed as IMA, therefore it has been widely discussed and concerned in recent years.^[3–12]^ With more and more observation and analysis of CMPT, it is found that the papillary structure, surface ciliated cells and mucinous cells are not absolutely necessary for the diagnosis. Some CMPT-like tumors may present flat growth pattern along the alveolar wall which were covered by Clara and/or alveolar cells on the surface. However, there was always a continuous basal cell layer at the bottom. Therefore, cases with papillary structures were named as classic CMPT, while those without papillary structures were proposed as non-classic CMPT.^[13]^ In order to solve this dilemma, Chang, et al carried a well-designed experimental observation, and found that the morphological change and immunophenotype of CMPT, both classic and non-classic types, are essentially consistent with the continuous spectrum of the components of mucous epithelial cells from the proximal to the distal bronchioles. So it is suggested to name these kinds of tumors as the diagnosis name of BA.^[1]^ This convincing work expanded the understanding of CMPT, and BA will be recognized in the upcoming World Health Organization classification in 2020.

Although the recognition of BA has made new progress, there are still some difficulties in its differential diagnosis. For example, the distal BA covered mainly by alveolar cells or Clara cells in the luminal layer needs to be differentiated from adenocarcinoma in situ. Vigorous proliferation of basal cells also needs to be discriminated from sclerosing pneumocytoma. The most challenging situation is the differentiation between BA rich in mucinous cells and IMA, especially in the rapid frozen pathological diagnosis, like the current case. Pathologists need to carefully look for cuboidal and/or ciliated cells to avoid the pitfall. If the luminal layer was purely lined by mucinous cells, pathologist must pay more effort to search areas showing bilayerd composition with the basal layer at the bottom. This is the key lesson we learned from the current case. Another 2 benign tumors, mucinous adenomas and mucinous cystadenomas, also need to be considered in differential diagnosis. These 2 tumors contain only a small number of myoepithelial cells at the bottom layer rather than a continuous basal cell layer, therefore, immunohistochemical staining of myoepithelial markers may contribute to the differential diagnosis.

Driver mutations have been observed in BAs.^[1,4,5,7,13,14]^ In the current study, we demonstrated that this special case harboring 2 different driver mutations simultaneously, which are rarely seen even in adenocarcinoma of the lung. It still needs further study to reveal whether coexistence of multiple driver-gene mutations may lead to worse prognosis.

In conclusion, this case expanded the understanding of BA that the luminal layer can be mucinouse cells alone without any other components, such as cilated cells, cuboid cells clara cells and/or alveolar cells.

## Author contributions

SL, EHW and LW analyzed the data and wrote the manuscript. MX performed the immunochemical staining. NL performed genetic analysis. All authors have read and approved the final manuscript.

## References

[1] Chang JC Montecalvo J Borsu L Bronchiolar adenoma: expansion of the concept of ciliated muconodular papillary tumors with proposal for revised terminology based on morphologic, immunophenotypic, and genomic analysis of 25 cases. Am J Surg Pathol 2018;42:1010–26. 29846186 10.1097/PAS.0000000000001086 PMC8063713

[2] Ishikawa Y Ciliated muconodular papillary tumor of the peripheral lung: benign or malignant? Pathol Clin Med (Byouri-to-Rinsho) 2002;964.

[3] Kamata T Yoshida A Kosuge T Ciliated muconodular papillary tumors of the lung: a clinicopathologic analysis of 10 cases. Am J Surg Pathol 2015;39:753–60. 25803171 10.1097/PAS.0000000000000414

[4] Udo E Furusato B Sakai K Ciliated muconodular papillary tumors of the lung with KRAS/BRAF/AKT1 mutation. Diagnost PatholV 12 2017;62. 10.1186/s13000-017-0651-2 PMC5568349 28830562

[5] Kamata T Sunami K Yoshida A Frequent BRAF or EGFR mutations in ciliated muconodular papillary tumors of the lung. J Thorac Oncol 2016;11:261–5. 26718882 10.1016/j.jtho.2015.10.021

[6] Sato S Koike T Homma K Ciliated muconodular papillary tumour of the lung: a newly defined low-grade malignant tumour. Interact Cardiovasc Thorac Surg 2010;11:685–7. 20724424 10.1510/icvts.2009.229989

[7] Jin Y Shen X Shen L Ciliated muconodular papillary tumor of the lung harboring ALK gene rearrangement: case report and review of the literature. Pathol Int 2017;67:171–5. 28150468 10.1111/pin.12512

[8] Chuang HW Liao JB Chang HC Ciliated muconodular papillary tumor of the lung: a newly defined peripheral pulmonary tumor with conspicuous mucin pool mimicking colloid adenocarcinoma: a case report and review of literature. Pathol Int 2014;64:352–7. 25047506 10.1111/pin.12179

[9] Kon T Baba Y Fukai I Ciliated muconodular papillary tumor of the lung: a report of five cases. Pathol Int 2016;66:633–9. 27671838 10.1111/pin.12460

[10] Hata Y Yuasa R Sato F Ciliated muconodular papillary tumor of the lung: a newly defined low-grade malignant tumor with CT findings reminiscent of adenocarcinoma. Jpn J Clin Oncol 2013;43:205–7. 23275641 10.1093/jjco/hys218

[11] Lau KW Aubry MC Tan GS Ciliated muconodular papillary tumor: a solitary peripheral lung nodule in a teenage girl. Hum Pathol 2016;49:22–6. 26826405 10.1016/j.humpath.2015.09.038

[12] Yao X Gong Y Zhou J A surgical case of ciliated muconodular papillary tumor. Thorac Cancer 2019;10:1019–22. 30788905 10.1111/1759-7714.12997 PMC6449262

[13] Zheng Q Luo R Jin Y So-called “non-classic” ciliated muconodular papillary tumors: a comprehensive comparison of the clinicopathological and molecular features with classic ciliated muconodular papillary tumors. Hum Pathol 2018;82:193–201. 30092236 10.1016/j.humpath.2018.07.029

[14] Kataoka T Okudela K Matsumura M A molecular pathological study of four cases of ciliated muconodular papillary tumors of the lung. Pathol Int 2018;68:353–8. 29624782 10.1111/pin.12664

